# Darifenacin treatment for overactive bladder in patients who expressed dissatisfaction with prior extended-release antimuscarinic therapy

**DOI:** 10.1111/j.1742-1241.2008.01893.x

**Published:** 2008-11

**Authors:** N Zinner, K C Kobashi, U Ebinger, A Viegas, M Egermark, E Quebe-Fehling, P Koochaki

**Affiliations:** 1 Western Clinical Research, Inc.Torrance, CA, USA; 2 Virginia Mason Medical CenterSeattle, WA, USA; 3 Novartis Pharmaceuticals CorporationEast Hanover, NJ, USA; 4 Novartis Pharma AGBasel, Switzerland; 5 Procter and Gamble Pharmaceuticals CorporationCincinnati, OH, USA

## Abstract

**Introduction and objective:**

Patient perception of overactive bladder (OAB) treatment outcomes can be a useful indicator of benefit and may help drive persistence on treatment, which is known to be poor in OAB. It remains unclear whether OAB patients dissatisfied with one antimuscarinic can achieve satisfaction with another and supporting data are limited. This study investigated patient-reported outcomes and clinical parameters during darifenacin treatment in OAB patients who expressed dissatisfaction with prior extended-release (ER) oxybutynin or tolterodine therapy (administered for ≥ 1 week within the past year).

**Methods:**

This open-label study was conducted in darifenacin-naïve OAB patients. Patients received 7.5 mg darifenacin once daily with the possibility of up-titrating to 15 mg after 2 weeks, for up to 12 weeks. Efficacy parameters included the Patient’s Perception of Bladder Condition (PPBC), patient satisfaction with treatment, micturition frequency and number of urgency and urge urinary incontinence (UUI) episodes. Adverse events (AEs) were also recorded.

**Results:**

In total, 497 patients were treated (84.1% women). Darifenacin treatment resulted in statistically significant improvements in PPBC scores, micturition frequency, urgency and UUI episodes from baseline at 12 weeks. The improvements were similar for patients previously treated with oxybutynin ER or tolterodine ER. More than 85% of patients expressed satisfaction with darifenacin. As noted in other studies, the most common AEs were dry mouth and constipation, but these infrequently resulted in treatment discontinuation, which was low overall.

**Conclusions:**

In this study, PPBC score and OAB symptoms were significantly improved, and satisfaction was high during treatment with darifenacin (7.5/15 mg) in patients who were dissatisfied with the previous antimuscarinic treatment.

**What's known**

- It has been suggested that treatment with the most commonly used antimuscarinic agents for overactive bladder, extended-and immediate-release (ER and IR) oxybutynin and tolterodine, may be unsuccessful because of issues with patient non-compliance and discontinuation before maximal therapeutic benefit can be achieved.
- Data demonstrating that patients dissatisfied with one particular OAB treatment may benefit from the use of another antimuscarinic are lacking.

**What's new**

- This is the first study to show that darifenacin treatment can be associated with significant improvements from baseline in OAB symptoms, patients’ perception of treatment outcome and treatment satisfaction in patients who had expressed dissatisfaction with prior ER antimuscarinic therapy.
- This study utilises a unique patient satisfaction scale, designed to resemble real-life questions posed by a physician.

## Introduction

Drug treatments for overactive bladder (OAB) may fail because of non-adherence or discontinuation. These issues are frequently observed in clinical practice and were exemplified in a recent evaluation of both the extended-release (ER) and immediate-release formulations of the most commonly used antimuscarinic agents for OAB, oxybutynin and tolterodine, in a regional managed healthcare plan. One of the conclusions was that persistence overall was low and more patient-reported data on outcomes of OAB drug therapy are needed to improve understanding of non-adherence and discontinuations (1). In particular, the patient's perception of effectiveness and tolerability may be a useful tool to predict whether patients stay on treatment or not.

Amongst newer antimuscarinic agents, darifenacin has been identified as having particularly high *in vitro* selectivity for the M_3_ muscarinic receptor subtype thought to be responsible for detrusor contraction (2,3). The efficacy, tolerability and safety of darifenacin in the treatment of OAB are well-established.

For some OAB patients, switching to another antimuscarinic could potentially achieve improvement in their symptoms. However, data demonstrating that patients already dissatisfied with one particular OAB treatment may benefit from the use of another agent are often lacking. Here, we evaluated patient reports of treatment benefit and satisfaction with treatment, clinical efficacy, tolerability and safety parameters during darifenacin treatment in an open-label, 12-week study in patients who had expressed dissatisfaction with efficacy and/or side effects with prior treatment with ER formulations of oxybutynin or tolterodine for OAB. These data are supplemented by a novel measure of patient satisfaction, a variable which, in clinical practice, can be directly related to patient adherence and persistence with therapy across diverse medical specialities.

## Materials and methods

### Study design

This was a 12-week, open-label, single-arm, multicentre study to evaluate the patient’s perception of treatment benefits with darifenacin treatment in patients with OAB who had expressed dissatisfaction with prior treatment with ER formulations of oxybutynin or tolterodine for reasons including insufficient efficacy and adverse events (AEs) or both.

The study (NCT00366002) was conducted in 78 centres in the USA between June 2006 and September 2007, in accordance with the International Conference on Harmonisation Harmonised Tripartite Guidelines for Good Clinical Practice, with applicable local regulations and with the ethical principles laid down in the Declaration of Helsinki. Written informed consent was obtained from each study participant.

### Patients

The study population consisted of men and women (≥ 18 years of age) with OAB symptoms [an average of ≥ 8 micturitions/24 h and ≥ 1 urgency episode/24 h, with or without urgency urinary incontinence (UUI) episodes] for at least 6 months prior to randomisation, and with a baseline score of ≥ 2 on the Patient Perception of Bladder Condition (PPBC) questionnaire at screening. To be included in the study, patients were required to be naïve to darifenacin treatment, to have received at least 1 week of treatment with oxybutynin ER or tolterodine ER within the year prior to this trial and to report that they were dissatisfied with the most recent of these treatments (assessed by response to a Patient’s Previous Treatment Assessment question at study visit 1). Patient retention on treatment was expected to be high because of their participation in an open-label trial.

Patients with a mean daily urinary volume > 3000 ml or a mean volume voided/micturition of > 300 ml, as verified in the micturition diary for two consecutive days prior to baseline, were excluded, as were those with clinically predominant and bothersome stress urinary incontinence, urinary retention, clinically significant bladder outlet obstruction, an indwelling catheter or intermittent self-catheterisation. Those with other significant medical problems or urogenital conditions, including neurogenic bladder, cystocele or distal pelvic organ prolapse, frequent urinary tract infections (≥ 3 over the preceding year) or urogenital surgery in the previous year or unexplained haematuria at screening, were also excluded. Participation in a bladder-training programme or any electro-stimulation therapy within 2 weeks prior to screening was also prohibited. Women of childbearing age were required not to be pregnant or nursing, and to be using acceptable methods of contraception.

Concomitant treatments were also restricted; the use of drugs known to affect mainly urinary bladder function (e.g. anticholinergics, antispasmodics, serotonin-noradrenaline-reuptake-inhibitors) was prohibited at any time during the study, but stable doses of alpha blockers or 5-alpha-reductase inhibitors were permitted for patients with benign prostatic hyperplasia. Treatments prohibited during the prescreening period included cholinergic agonists, cholinesterase inhibitors (e.g. bethanecol, donepezil and rivastigmine), potent inhibitors of cytochrome CYP3A4 (e.g. ketoconazole, itraconazole, ritonavir, nelfinavir, clarithromycin and nefazadone), potent P-glycoprotein inhibitors (e.g. cyclosporine and verapamil), drugs with significant anticholinergic side effects (e.g. tricyclic antidepressants, selective-serotonin-reuptake-inhibitors and first generation antihistamines) or any other investigational drug.

### Treatment and assessments

**Figure 1:**
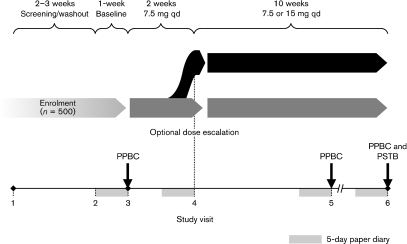
Study design overview. qd, once daily; PPBC, Patient Perception of Bladder Condition; PSTB, Patient Satisfaction with Treatment Benefits

After a prescreening/washout period during which prohibited medications were discontinued (at study visit 2), patients were asked to complete a bladder diary for 5 consecutive days to establish baseline symptoms and OAB severity during the 7 days prior to the baseline visit (visit 3). Eligible patients then received darifenacin 7.5 mg once daily (qd) for the first 2 weeks postbaseline; subsequently (at visit 4) voluntary up-titration to darifenacin 15 mg qd was permitted if the patient required additional efficacy, and treatment was well tolerated. Down-titration of darifenacin was not permitted. The remaining patients received darifenacin 7.5 mg qd throughout the study (Figure 1).

The primary efficacy variable was the change from baseline to study end (visit 6) in the score on the PPBC, a validated six-point categorical Likert scale questionnaire (4) in which patients select one of six categories that best describe their bladder condition, ranging from ‘does not cause me any problems at all’ to ‘causes me many severe problems’. Patients completed the PPBC questionnaire at baseline (visit 3) and after 6 and 12 weeks of treatment (at visits 5 and 6 respectively).

Patients also completed an exploratory questionnaire developed for this study, the Patient Satisfaction with Treatment Benefit (PSTB) questionnaire, after 12 weeks of treatment (visit 6). Prior to being implemented in the study, the PSTB underwent pilot testing in which it was shown to contain items that are clearly understood and relevant to OAB sufferers. Part I of the PSTB assesses overall satisfaction with the treatment benefits received from therapy using a five-point Likert scale, with five response options ranging from ‘extremely satisfied’ to ‘not at all satisfied’. Part II of the PSTB contains 23 items that assess patient satisfaction with treatment for improving the effects of OAB on their sleep, daily activities, emotions and reducing the level of coping behaviours (e.g. pad usage), using the same five-point Likert scale, with an additional response option of ‘not applicable’.

Efficacy was also evaluated relative to baseline after 2, 6 and 12 weeks of treatment (at visits 4, 5 and 6 respectively) using data on micturition frequency, urgency and UUI episodes collected in a 5-day bladder symptom paper diary over five consecutive days during the 7 days prior to the study visit. UUI episodes were recorded by checking both urinary leak and urgency. Tolerability and safety were monitored throughout the trial based on AEs, treatment discontinuations, vital signs and laboratory parameters recorded at each study visit.

### Statistical analysis

The intent-to-treat population comprised all patients who received at least one dose of study drug. A study sample size of 398 was calculated to have 90% power to detect a significant change from baseline in PPBC score, using Wilcoxon’s signed-rank test and a two-sided 5% significance level. Allowing for a 20% withdrawal rate, 500 patients were calculated to be required for the study.

The change from baseline to study end (visit 6) in PPBC (primary efficacy variable) was analysed by Wilcoxon’s signed-rank test for all patients in the intent-to-treat population who responded to the PPBC questionnaire both at baseline (visit 3) and postbaseline (visits 5 and/or 6). If visit 6 data were missing, visit 5 data were carried forward (if available). Odds for improvement in PPBC score were calculated as the ratio between the proportion of patients with an improvement and those without improvement, and used to calculate the ratio between odds for improvement in the two previous treatment groups (oxybutynin ER vs. tolterodine ER). Logistic regression analysis of the resulting odds ratio was used to detect differences between groups. These analyses were also conducted *post hoc* for the subset of patients who were receiving oxybutynin ER or tolterodine ER at screening.

Secondary patient-reported outcome variables included the change in PPBC from baseline to visit 5, and the percentage of patients categorised as having ‘improvement’, ‘no change’ and ‘deterioration’ at both visits 5 and 6, together with the corresponding 95% confidence intervals (CI). The percentage of patients categorised as ‘satisfied’ based on their responses to the PSTB (part I) was also calculated and explored by a logistic regression model adjusting for age, gender and stratum (previous treatment with oxybutynin ER or tolterodine ER).

Clinical symptom outcomes based on bladder diary responses (number of micturitions/day, urgency episodes/week and UUI episodes/week) were analysed by Wilcoxon’s signed-rank test at visits 5 and 6 and by Poisson regression adjusting for baseline, age, gender and previous treatment. Patients without UUI episodes at baseline were not included in the analysis of UUI episodes. In addition, a *post hoc* analysis was conducted to evaluate the magnitude of change from baseline to study end in bladder diary parameters (micturition frequency, urgency and UUI episodes) in patients reporting a change in PPBC score of 0, 1, 2 or 3. AEs were summarised descriptively for the safety population, comprising all patients who received at least one dose of darifenacin. The results are presented here for all prespecified variables included in the statistical analysis plan and the *post hoc* analyses mentioned.

## Results

### Patients

Of 500 patients enrolled, 497 patients received study drug and comprised the intent-to-treat and safety populations of the study. This included 218 patients previously treated with oxybutynin ER and 279 previously treated with tolterodine ER, of whom approximately 34% (75/218 and 95/279 respectively) were still receiving this treatment at the screening visit. These two subsets were comparable in baseline demographics with the overall study population (Table 1). There was no evidence of UUI episodes in 17.1% of all patients at baseline.

**Table 1:** Baseline demographics and characteristics for the study populations according to the previous OAB treatment

	Previous treatment		
Parameter	Oxybutynin ER	Tolterodine ER	All patients
*Total study population*	*n*= 218	*n*= 279	*n*= 497
**Age**			
Mean ± SD, years	61.3 ± 12.8	60.6 ± 12.7	60.9 ± 12.7
Range, years	25–89	24–84	24–89
≥ 65 years, *n* (%)	97 (44.5)	114 (40.9)	211 (42.5)
Women, *n* (%)	178 (81.7)	240 (86.0)	418 (84.1)
**Race, *n* (%)**			
Caucasian	202 (92.7)	255 (91.4)	457 (92.0)
Black	10 (4.6)	20 (7.2)	30 (6.0)
Other	6 (2.8)	4 (1.4)	10 (2.0)
**Reason for dissatisfaction with the previous treatment**			
Intolerable side effects	20 (9.2)	27 (9.7)	47 (9.5)
Insufficient efficacy	166 (76.1)	217 (77.8)	383 (77.1)
Both insufficient efficacy and intolerable side effects	32 (14.7)	35 (12.5)	67 (13.5)
**OAB parameters, median**			
Micturitions/day	10.2	10.4	10.2
Urgency episodes/day	5.6	5.4	5.6
UUI episodes/week	16.8	16.8	16.8
*Patients receiving prior ER antimuscarinic therapy at screening*	*n*= 75	*n*= 95	*n*= 170
**Age**			
Mean ± SD, years	63.3 ± 12.5	61.5 ± 12.6	62.3 ± 12.5
Range, years	32–85	31–84	31–85
≥ 65 years, *n* (%)	40 (53.3)	41 (43.2)	81 (47.6)
Women, *n* (%)	60 (80.0)	82 (86.3)	142 (83.5)
**Race, *n* (%)**			
Caucasian	72 (96.0)	86 (90.5)	158 (92.9)
Black	2 (2.7)	7 (7.4)	9 (5.3)
Other	1 (1.3)	2 (2.1)	3 (1.8)

ER, extended-release; OAB, overactive bladder; PPBC, patient perception of bladder condition; SD, standard deviation; UUI, urgency urinary incontinence.

**Figure 2:**
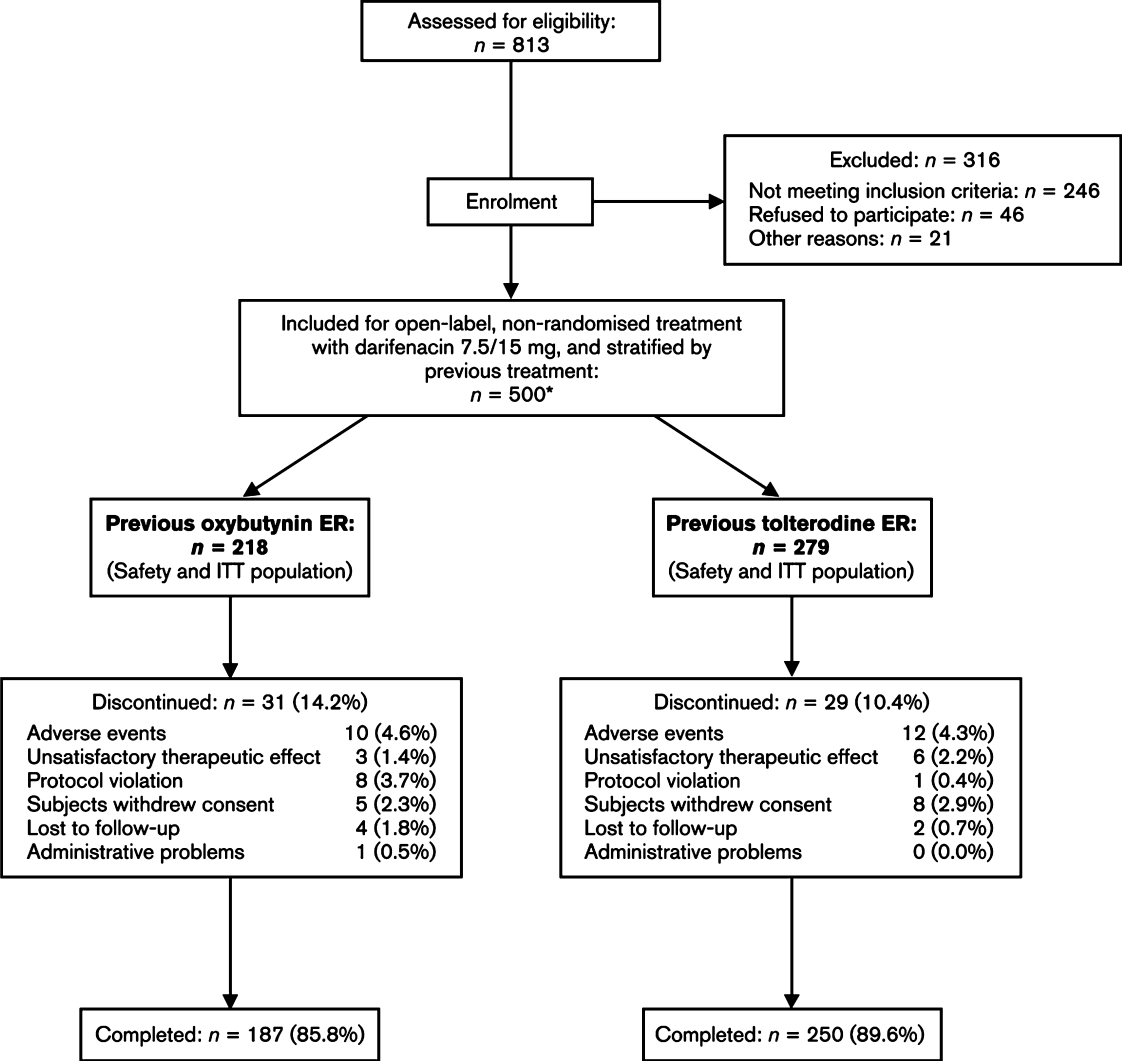
Subject disposition and flow. ER, extended-release; ITT, intent-to-treat. *Three patients were included but did not receive treatment

A total of 60 patients discontinued the study prematurely (31 previously treated with oxybutynin ER and 29 previously treated with tolterodine ER). The main reasons for discontinuation were AEs (4.4%), withdrawal of consent (2.6%), protocol deviation (1.8%) and unsatisfactory therapeutic effect (1.8%), and were comparable between patients previously treated with oxybutynin ER or tolterodine ER except for the number of protocol deviations (eight patients previously treated with oxybutynin ER vs. one previously treated with tolterodine ER). Patient disposition is summarised in Figure 2.

The darifenacin dose was increased to 15 mg qd for 62.6% of the patients remaining in the study at 2 weeks (302/482), comprising 65.9% (137/208) previously treated with oxybutynin ER and 60.2% (165/274) previously treated with tolterodine ER.

### Efficacy: patient-reported outcomes

The PPBC scores for the change in bladder condition from baseline (Table 2) were significantly reduced by 12 weeks of darifenacin treatment (p < 0.0001). Almost identical, statistically significant findings were observed in a *post hoc* analysis of patients still receiving their previous antimuscarinic therapy at screening (p < 0.0001). This level of improvement was also achieved after 6 weeks of treatment (visit 5), with a median score of 3.0, and a mean ± standard deviation (SD) score of 3.1 ± 1.20 (p < 0.0001 vs. baseline). At both 6 and 12 weeks, the median change in PPBC from baseline was −1.0 and the mean change was −1.4. This corresponds to a change on the PPBC scale from ‘my bladder causes me some moderate/severe problems’ (baseline mean score = 4.5) to ‘my bladder causes me some minor problems’ (visit 6 mean score = 3.1).

**Table 2:** Influence of up to 12 weeks of flexible-dose darifenacin treatment on PPBC and clinical symptoms

	Previous treatment		
	Oxybutynin ER (*n*= 218)	Tolterodine ER (*n* = 279)	All patients (*n*= 497)
**PPBC**			
*Total study population*			
Patients, *n*	214	275	489
Baseline			
Mean ± SD	4.6 ± 0.79	4.5 ± 0.76	4.5 ± 0.77
Median	5.0	4.0	4.0
12 weeks			
Mean ± SD	3.1 ± 1.24	3.1 ± 1.27	3.1 ± 1.25
Median	3.0	3.0	3.0
Change			
Mean ± SD	−1.4 ± 1.26	−1.4 ± 1.33	−1.4 ± 1.30
Median	−1.0	−1.0	−1.0
p-value*	< 0.0001	< 0.0001	< 0.0001
*Patients receiving prior ER antimuscarinic therapy at screening*			
Patients, *n*	75	94	169
Baseline			
Mean ± SD	4.6 ± 0.85	4.6 ± 0.82	4.6 ± 0.84
Median	5.0	5.0	5.0
12 weeks			
Mean ± SD	3.2 ± 1.26	2.9 ± 1.31	3.0 ± 1.29
Median	3.0	3.0	3.0
Change			
Mean ± SD	−1.4 ± 1.41	−1.6 ± 1.34	−1.5 ± 1.37
Median	−1.0	−2.0	−1.0
p-value*	< 0.0001	< 0.0001	< 0.0001
**Micturitions/day**			
Patients, *n*	208	274	482
Baseline			
Mean ± SD	10.9 ± 2.90	10.8 ± 2.60	10.8 ± 2.73
Median	10.2	10.4	10.2
12 weeks			
Mean ± SD	8.9 ± 2.60	8.4 ± 2.56	8.6 ± 2.59
Median	8.4	8.2	8.2
Change			
Mean ± SD (absolute)	−2.0 ± 2.23	−2.2 ± 2.50	−2.2 ± 2.39
Median (absolute)	−1.8	−2.0	−2.0
Median (%)	−17.4	−21.2	−19.5
p-value*	< 0.0001	< 0.0001	< 0.0001
**Urgency episodes/day**			
Patients, *n*	208	274	482
Baseline			
Mean ± SD	6.3 ± 3.41	6.2 ± 3.30	6.2 ± 3.34
Median	5.6	5.6	5.6
12 weeks			
Mean ± SD	3.4 ± 3.45	3.0 ± 3.34	3.2 ± 3.39
Median	2.2	1.8	1.8
Change			
Mean ± SD (absolute)	−2.8 ± 2.97	−3.2 ± 3.35	−3.1 ± 3.20
Median (absolute)	−2.6	−3.0	−2.8
Median (%)	−55.5	−64.0	−61.6
p-value*	< 0.0001	< 0.0001	< 0.0001
**UUI episodes/week**			
Patients, *n*	175	227	402
Baseline			
Mean ± SD	16.7 ± 14.81	16.5 ± 16.15	16.6 ± 15.56
Median	11.2	11.2	11.2
12 weeks			
Mean ± SD	6.8 ± 11.49	5.0 ± 9.73	5.8 ± 10.56
Median	2.8	1.4	1.4
Change			
Mean ± SD (absolute)	−9.9 ± 13.89	−11.5 ± 13.25	−10.8 ± 13.54
Median (absolute)	−8.4	−8.4	−8.4
Median (%)	−83.3	−88.0	−85.7
p-value*	< 0.0001	< 0.0001	< 0.0001

*p-value for absolute (PPBC) or % change from baseline using the Wilcoxon signed-rank test. Data at 12 weeks were calculated using last-observation carried forward method. ER, extended-release; PPBC, Patient Perception of Bladder Condition; SD, standard deviation; UUI, urgency urinary incontinence.

The effect of darifenacin in improving PPBC was evident regardless of previous treatment (Table 2). In a logistic regression analysis, the odds (and 95% CI) for improvement in PPBC amongst previous recipients of oxybutynin ER or tolterodine ER were 2.08 (1.48, 2.92) and 1.77 (1.29, 2.43) respectively. There was no statistically significant difference between the two prior treatment groups on PPBC, with an odds ratio for improvement in oxybutynin: tolterodine recipients of 1.17 (95% CI: 0.78, 1.77).

In line with these findings, evaluation of individual responses indicated that most patients (356/497, 72.8%) reported an improvement in PPBC, with 113 (23.1%) reporting no change after 12 weeks of treatment, and 20 patients (4.1%) reporting an increase in their PPBC score (i.e. symptom deterioration). In addition, 110 of the 230 patients (47.8%) reporting ‘severe’ or ‘many severe’ problems on the PPBC questionnaire at baseline shifted to ‘does not cause me any problems at all’, ‘causes me minor problems’ or ‘causes me some minor problems’ after 12 weeks of treatment with darifenacin.

Assessment of overall satisfaction with treatment benefit using the PSTB showed that 405 patients (85.6%) were ‘satisfied’ with darifenacin treatment after 12 weeks, and 68 (14.4%) were ‘not satisfied’. Satisfaction levels were not statistically significantly influenced by previous treatment: odds for reporting satisfaction (and 95% CI) were 4.35 (2.90, 6.53) amongst previous oxybutynin ER recipients and 5.23 (3.50, 7.80) for tolterodine ER recipients, representing an odds ratio (95% CI) of 0.83 (0.50, 1.40). Similarly, satisfaction with individual aspects of OAB condition in part II of the PSTB questionnaire after 12 weeks was evident, with only minor differences according to previous treatment received (data not shown).

### Efficacy: bladder diary parameters

**Figure 3:**
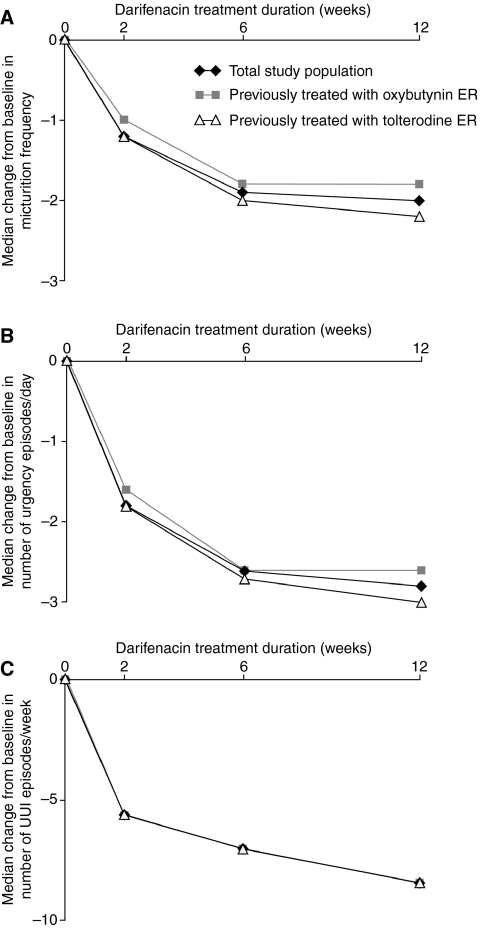
Efficacy of darifenacin treatment on OAB symptoms in the intent-to-treat population (LOCF analysis), seen as: (A) daily micturition frequency, (B) daily urgency episodes and (C) weekly UUI episodes. ER, extended-release; UUI, urgency urinary incontinence

Darifenacin treatment for 12 weeks resulted in significant improvements in micturition frequency, urgency episodes and UUI episodes compared with baseline, both for the overall study population and after stratification by prior treatment (all p < 0.0001) (Table 2). Improvements in these OAB symptoms reached statistical significance within 2 weeks of initiating darifenacin treatment and appeared to improve further after 6 and 12 weeks (Figure 3). An informal *post hoc* analysis indicated that the reductions in each of the three micturition variables in the overall study population at study end were consistently associated with increasing degree of improvement in PPBC scores.

In a Poisson regression analysis, after adjustment for baseline, gender, age and previous therapy, the efficacy of darifenacin on micturition frequency did not differ significantly between patients according to previous oxybutynin ER or tolterodine ER treatment. However, compared with patients previously given oxybutynin ER, patients previously given tolterodine ER experienced significantly fewer urgency episodes at study end (oxybutynin vs. tolterodine: adjusted mean 3.4 vs. 3.0, median 2.2 vs. 1.8, p = 0.0296) and significantly fewer UUI episodes after 2 weeks (adjusted mean 8.1 vs. 7.9, median 4.2 vs. 3.5, p = 0.0426), 6 weeks (adjusted mean 6.1 vs. 4.9, median 2.8 vs. 1.4, p < 0.0001) and 12 weeks of darifenacin treatment (adjusted mean 6.8 vs. 5.0, median 2.8 vs. 1.4, p < 0.0001) respectively.

**Figure 4:**
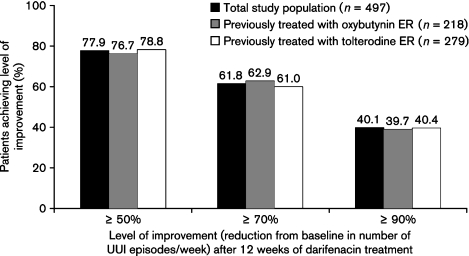
Different response levels for patients with ≥ 7 UUI episodes per week at baseline after 12 weeks of darifenacin treatment, according to previous treatment received. ER, extended-release; UUI, urgency urinary incontinence

Further investigation indicated that this apparently greater efficacy of darifenacin on UUI episodes in patients previously treated with tolterodine ER also occurred in patients with more severe OAB at baseline (i.e. those reporting at least seven UUI episodes per week at baseline). Amongst such patients previously given oxybutynin ER (*n* = 129) vs. tolterodine ER (*n* = 159), the number of UUI episodes was relatively high after 2 weeks on darifenacin 7.5 mg (adjusted mean 10.3 vs. 10.7, median 5.6 vs. 7.0); UUI episodes subsequently decreased to 7.9 vs. 6.6 (4.2 vs. 2.8) after 6 weeks and 8.6 vs. 6.8 (2.8 vs. 2.8) after 12 weeks, respectively. However, there were no significant differences according to prior treatment in darifenacin response rates at any cut-off rate, i.e. the proportion of patients achieving at least 50%, 70% or 90% reduction in UUI episodes per week (Figure 4).

The response to darifenacin treatment in terms of dry days was similar between prior oxybutynin ER and tolterodine ER recipients, with 64.2% vs. 64.5% of patients achieving at least three consecutive dry days respectively. The data suggested that more tolterodine ER-than oxybutynin ER-pretreated patients achieved a state approaching continence and bladder control after 12 weeks of treatment with darifenacin, although the clinical relevance of this finding remains to be determined. At study end, 44.2% vs. 51.0% previous oxybutynin ER vs. previous tolterodine ER recipients achieved at least five consecutive dry days on darifenacin and 40.6% vs. 48.2% achieved < 8 voids per day.

### Tolerability and safety

The most common AEs during darifenacin treatment were dry mouth (reported by 20.1% of patients) and constipation (14.1%; Table 3). Both events were reported slightly less frequently by patients previously treated with oxybutynin ER (16.1% dry mouth and 11.0% constipation) than tolterodine ER (23.3% and 16.5% respectively). These AEs were well tolerated, infrequently leading to treatment discontinuation; only 10 (4.6%) and 12 (4.3%) patients previously treated with oxybutynin ER or tolterodine ER discontinued darifenacin treatment because of an AE, respectively, and overall only 1.6% of patients discontinued because of dry mouth and 0.8% because of constipation. There were no deaths during the study and no clinically relevant changes in laboratory parameters or vital signs. Seven patients experienced serious AEs, none of which was considered treatment related by the investigator.

**Table 3:** Most frequent adverse events (≥ 2% of total population)

Preferred term	Previous oxybutynin ER, *N*= 218, *n* (%)	Previous tolterodine ER, *N*= 279, *n* (%)	Total, *N*= 497, *n* (%)
Any AE(s)	119 (54.6)	171 (61.3)	290 (58.4)
Dry mouth	35 (16.1)	65 (23.3)	100 (20.1)
Constipation	24 (11.0)	46 (16.5)	70 (14.1)
Urinary tract infection	12 (5.5)	21 (7.5)	33 (6.6)
Headache	10 (4.6)	8 (2.9)	18 (3.6)
Nausea	5 (2.3)	11 (3.9)	16 (3.2)
Dyspepsia	5 (2.3)	8 (2.9)	13 (2.6)
Dry eye	2 (0.9)	9 (3.2)	11 (2.2)
Upper respiratory tract infection	5 (2.3)	5 (1.8)	10 (2.0)

AEs, adverse events.

## Discussion

In this study involving patients who expressed dissatisfaction with prior OAB treatment with oxybutynin ER or tolterodine ER because of insufficient efficacy or tolerability problems or both, open-label darifenacin administration for 12 weeks resulted in improvements in patient perceptions of their bladder condition that correlated with improvements in clinical symptoms (micturition frequency, urgency and UUI episodes). These improvements were accompanied by a favourable tolerability profile, resulting in a large majority of patients (approximately 86%) reporting satisfaction with darifenacin treatment.

The improvements observed in this study were comparable to those previously reported in other studies with antimuscarinic agents. For example, the mean PPBC score decreased from baseline 4.6 to 3.1 after 12 weeks of darifenacin treatment in the present study, and from 4.4 to 2.9 after 12 weeks of solifenacin treatment in the VOLT study (5). In addition, the reductions in micturition frequency, urgency and UUI episodes were comparable to those seen in the previous darifenacin studies using the same dosing regimen (6,7). OAB symptom improvements in our study were also comparable to the findings reported in the VESlcare Efficacy and Research Study US (VERSUS) study, in which patients dissatisfied with tolterodine treatment were switched to solifenacin (8), and a long-term extension study in which patients were permitted to switch to open-label solifenacin following double-blind tolterodine treatment (9).

The incidence and nature of AEs reported in this study were consistent with those reported in earlier clinical trials with darifenacin. These results are further supported by high levels of persistence with darifenacin therapy and well-maintained treatment benefit in previous long-term studies (over 2 years in duration) (10,11).

This open-label study also evaluated levels of patient satisfaction via the use of a novel exploratory satisfaction scale (PSTB questionnaire), which was designed to resemble real-life questions posed by a physician. Patients were required to recall their experiences of previous antimuscarinic treatment at baseline, while PSTB responses were recorded at the end of darifenacin treatment to determine whether these patients expressing dissatisfaction with their previous antimuscarinic achieved satisfaction with darifenacin. PSTB results indicated that more than 85% of patients expressed satisfaction with darifenacin treatment.

Patient satisfaction can be regarded as an important indicator of the quality of care across diverse medical specialities. Satisfaction with treatment is influenced by a combination of many factors, which go beyond traditional measures assessed in clinical trials (i.e. improvement in symptoms, and the type, frequency and severity of side effects). Interest in patient perceptions of the efficacy of their treatment has grown in recent years, resulting in the recent development by the Food and Drug Administration (FDA) of guidance for the pharmaceutical industry on the assessment of patient-reported outcomes in clinical trials (12). Despite this, a consensus on the best approach to assessing treatment satisfaction in clinical practice is yet to be reached. This study presents a novel method to assess patient satisfaction directly, which takes into account the real-life clinical situations. In the majority of patients (65.8%), previous treatment was still ongoing at the time of study entry, but in the remaining cases, previous treatment could have been discontinued up to a year before study entry. In clinical practice, visits to the physician commonly occur at intervals of 6–8 weeks or longer, and consequently, the physician may not see the patient within 1 week after a patient’s decision to terminate treatment (the period recommended for FDA analysis). Under these circumstances, the physician must rely on the patient’s recall of his or her treatment satisfaction, in a similar manner to the conduct of the present study. Similarly, the PSTB questionnaire provided a snapshot of patient perceptions of darifenacin treatment at study end, resembling the situation encountered in routine physician visits. However, administering the questionnaire during a single visit does not permit assessment of change in treatment perception over time (or between one treatment and the next), and this novel scale would benefit from further validation.

There are some weaknesses in this study that should be noted. Patient satisfaction seen in this study may reflect a response to a change in treatment, rather than the specific drug *per se*, coupled with satisfaction arising from a perceived improvement in overall care and increased monitoring associated with participation in a clinical study. The drug costs of the previous treatments may also have contributed to the lower satisfaction with the previous therapy, compared with free treatment provided during clinical trial participation. A longitudinal study design with consecutive treatments and accurate recording of patient perceptions of outcomes immediately after terminating each treatment would be required to address accurately the question whether more patients are satisfied following darifenacin treatment than with their previous treatment. As the discontinuation of prior ER antimuscarinic treatment could have taken place up to 1 year before entry into the present study, patient recall of satisfaction with the prior therapy may be unreliable. However, despite the low numbers of patients still receiving their previous antimuscarinic therapy at study entry (before washout), primary outcomes in this subset and the total study population were almost identical. It would also be of interest to determine whether patients switching to darifenacin remain satisfied with continued treatment. Similarly, further studies are warranted to determine whether the proportion of satisfied patients and the duration of satisfaction differ between antimuscarinic treatments.

The PSTB was developed with patient input and piloted with patients to ensure that patients understood the questionnaire items and that the items measured parameters that were relevant to patients. However, it has not been thoroughly validated as per the FDA draft guidance for developing new patient-reported outcomes in clinical trials (12). Further research is needed to establish the validity and reliability of the PSTB.

Patient-reported outcome measures are increasingly being viewed as important measures of treatment success, and indeed some have been described as better than objective measures (such as urodynamic testing) for gauging treatment efficacy (13,14). The present study utilised both a well-known, validated instrument (the PPBC) and a novel exploratory questionnaire (the PSTB), with both tools indicating significant levels of benefit from darifenacin treatment relative to baseline, regardless of the previous treatment received. These tools are particularly important for observing outcomes from the patient’s perspective, as they reflect subjective views of treatment impact on all aspects of daily living and individuals vary substantially in their desired outcomes and priorities or goals of treatment.

In conclusion, the findings of this study indicate that darifenacin 7.5/15 mg qd treatment can be associated with significant improvements from baseline in OAB symptoms and patient's perception of treatment outcome in patients who have expressed dissatisfaction with prior ER antimuscarinic therapy. Thus, darifenacin provides a valuable treatment option for such patients.
